# Identification of Kininogen-1 as a Serum Biomarker for the Early Detection of Advanced Colorectal Adenoma and Colorectal Cancer

**DOI:** 10.1371/journal.pone.0070519

**Published:** 2013-07-23

**Authors:** Jing Wang, Xinying Wang, Shiyong Lin, Chudi Chen, Congrong Wang, Qunying Ma, Bo Jiang

**Affiliations:** 1 Department of Gastroenterology, Nanfang Hospital, Southern Medical University, Guangzhou, Guangdong, China; 2 Guangdong Provincial Key Laboratory of Gastroenterology, Guangzhou, China; 3 Department of Endoscopy and Laser, Sun Yat-Sen University Cancer Center, Guangzhou, Guangdong, China; 4 Department of Laboratory Medicine, Nanfang Hospital, Southern Medical University, Guangzhou, Guangdong, China; Okayama University, Japan

## Abstract

**Background:**

Serum markers represent potential tools for the detection of colorectal cancer (CRC). The aim of this study was to obtain proteomic expression profiles and identify serum markers for the early detection of CRC.

**Methods:**

Proteomic profiles of serum samples collected from 35 healthy volunteers, 35 patients with advanced colorectal adenoma (ACA), and 40 patients with CRC were compared using Clinprot technology. Using enzyme-linked immunosorbent assays (ELISAs), 366 sera samples were additionally analyzed, and immunohistochemistry studies of 400 tissues were used to verify the expression of kininogen-1 and its value in the early detection of CRC.

**Results:**

Predicting models were established among the three groups, and kininogen-1 was identified as a potential marker for CRC using Clinprot technology. ELISAs also detected significantly higher serum kininogen-1 levels in ACA and CRC patients compared to controls (*P*<0.05). Furthermore, the area under the receiver operating characteristic curve (AUC) for serum kininogen-1 in the diagnosis of ACA was 0.635 (P = 0.003), and for serum carcinoembryonic antigen (CEA) was 0.453 (P = 0.358). The sensitivity, specificity, and accuracy of serum kininogen-1 for diagnosing Duke’s stage A and B CRC was 70.13%, 65.88%, and 67.90%, respectively, whereas serum CEA was 38.96%, 85.88%, and 63.58%, respectively. Moreover, immunohistochemistry showed that expression of kininogen-1 was significantly higher in CRC and ACA tissues than in normal mucosa (48.39% vs. 15.58% vs. 0%, *P*<0.05).

**Conclusions:**

These results suggest that Clinprot technology provides a useful tool for the diagnosis of CRC, and kininogen-1 is a potential serum biomarker for the early detection of advanced colorectal adenoma and CRC.

## Introduction

It is estimated that more than 143,000 individuals in the United States will be diagnosed with colorectal cancer (CRC) in 2012, and more than 50,000 individuals will die of this disease [1]. By screening average-risk individuals, it is hypothesized that CRC could be detected in its early stages, thereby reducing the rates of mortality associated with CRC [2]–[4]. Currently, CRC screening can include a fecal occult blood test, sigmoidoscopy, and colonoscopy [5]. In many resource-limited countries, the fecal occult blood test is primarily used, despite the insufficient sensitivity of this assay [6], [7]. Moreover, with sigmoidoscopy and colonoscopy being invasive and inconvenient procedures, their application for CRC screenings has been limited [8]. Therefore, less-invasive and non-invasive approaches are needed to improve sensitivity and patient compliance in CRC screenings.

The identification of serologic biomarkers specific for CRC could provide a relatively non-invasive and economically advantageous method for the detection of CRC compared to current screening options. However, serum is a complex body fluid that contains many diverse proteins. For example, more than 10,000 different proteins have been detected in human serum, and many proteins are secreted or shed by cells during different physiological or pathological processes [9], [10]. Therefore, it is difficult to identify a disease-specific serum marker. Due to advances in proteomics methodologies, it is now possible to rapidly identify novel candidate markers for cancer. For example, many studies have demonstrated the capacities for matrix-assisted laser desorption/ionization time-of-flight mass spectrometry (MALDI-TOF-MS) to separate complex mixtures of proteins, thereby facilitating a comparison of variations in protein expression for normal and cancerous serum samples [11]–[13]. In particular, Clinprot technology, which is based on MALDI-TOF-MS, has many advantages for clinical application, including its sensitivity, ease of use, and capacity for high-throughput analysis [14], [15].

Using MALDI-MS, Seraglia *et al.* [16] previously reported kininogen-1 to be a potentially novel plasma marker for CRC. Kininogen-1 is a multifunctional protein that plays an important role in many pathophysiological processes [17], including fibrinolysis, thrombosis, and inflammation, as well as having a role in oncogenesis [18]. To confirm these results and to identify additional novel plasma markers for CRC, sera from patients with CRC, patients with advanced colorectal adenoma (ACA), and healthy individuals were analyzed using Clinprot technology, enzyme-linked immunosorbent assays (ELISAs), and immunohistochemistry.

## Materials and Methods

### Patient Selection

All samples were collected from the Nanfang Hospital. Sera were collected between October 1, 2009 and December 31, 2010. Paraffin-embedded tissues were collected between April 1, 1999 and December 31, 2007. Patients with a known history of familial adenomatous polyposis, hereditary nonpolyposis colorectal cancer, hypertension, any other tumors, and obvious inflammatory diseases, were excluded. The following factors were recorded for each tissue: patient age, patient gender, tumor size, tumor location for CRC and ACA, tissue histology and tumor grade for intraepithelial neoplasia for ACA, differentiation, Duke’s stage, and Tumor-Node-Metastasis (TNM) stage (7th edn) for CRC. Colorectal adenoma patients having at least one adenoma ≥10 mm, or having a villous structure or carcinoma *in situ*, were classified as ACA patients [19].

This study was performed in accordance with institutional ethical guidelines and was approved by the Medical Ethics Committee of Southern Medical University (#2011119). Written informed consent forms were obtained from all patients.

### Sample Preparation

Sera were collected from healthy volunteers, ACA patients, preoperative CRC patients (obtained prior to any clinical treatment), and postoperative CRC patients (obtained seven days after surgery). For Clinprot analysis, a total of 110 sera samples were obtained from healthy volunteers (n = 35), ACA patients (n = 35), and preoperative CRC patients (n = 40). For ELISAs, a total of 366 sera were obtained from an additional 85 healthy volunteers, 80 ACA patients, 143 preoperative CRC patients, and 58 postoperative CRC patients. Asymptomatic and apparently healthy volunteers were selected that did not have a previous history of cancer. All samples were collected in 5 ml serum separator tubes, and then were incubated at RT for 30 min. Following centrifugation at 3000 rpm for 10 min, sera were distributed into 50 µl aliquots and stored at −70°C until needed.

A total of 400 paraffin-embedded tissues were analyzed by immunohistochemistry, including 75 normal colorectal mucosa tissues which were collected from healthy volunteers who underwent an endoscopic mucosal biopsy. In addition, 77 ACA tissues were collected from ACA patients who underwent endoscopic resection, and 248 CRC tissues were collected from CRC patients who underwent surgery. All specimens were fixed in 10% formalin solution and embedded in paraffin. Sections (4 µm) were cut and prepared for hematoxylin-eosin staining and immunohistochemistry assays.

### Extraction of Peptides from Serum Using Magnetic Beads, Followed by MALDI-TOF-MS Analysis

Serum peptides were separated and purified using a magnetic bead-based weak cation exchange chromatography purification kit (Bruker Daltonics GmbH). A total of 110 sera samples were fractionated according to the standard protocol proposed by the manufacturer. Strict quality control was maintained to ensure the accuracy and reproducibility of the results obtained. Serum peptide profiles were subsequently analyzed using an Ultraflex MALDI-TOF/TOF-MS (Bruker Daltonik, Bremen, Germany). Spectra were acquired in the mass/charge (m/z) range of 1,000 to 10,000 using FlexAnalysis software (Bruker Daltonics).

Clinpro Tools software 2.2 (Bruker, Daltonik) was used for the analysis of all data derived from serum samples. This included raw data obtained prior to treatment, baseline subtraction of spectra, normalization of spectra, internal peak alignment using prominent peaks, and a peak picking procedure. Moreover, pretreated data were visualized and statistically analyzed using Student’s t-test and genetic algorithms (GA). In addition, prediction models were established using the GA. To determine the accuracy of the prediction models generated, cross-validation was performed. Briefly, all samples were selected as a training set in the class predictor algorithm, then the same samples except for one randomly selected sample were used as a test set. A ‘test’ was then performed ten times randomly.

### Peptide Identification by MALDI-TOF/TOF

After completing the statistical analysis, differentially expressed peptides were identified. Initially, the mono-isotopic masses of peptides in the m/z range of 1,000 to 3,000 were determined using a reflective mode. After MS/MS spectra of precursor ions were acquired in the TOF/TOF mode, MS/MS data were subjected to a Mascot database search to identify the corresponding full-length protein matches.

### Enzyme-linked Immunosorbent Assay (ELISA)

All sera were analyzed in a blinded fashion, and standards and samples were run in triplicate. Kininogen-1 concentrations were quantified using a Human Kininogen ELISA Kit (No. EK2001-1, Assaypro, MO, USA). Briefly: (1) 25 µl standard or sample and 25 µl biotinylated kininogen was added to 96-well polystyrene microplates coated with a polyclonal antibody against human kininogen. After 2 h at RT, plates were washed five times, then 50 µl streptavidin-peroxidase conjugate was added to each well. After 30 min at RT, plates were washed five times and 50 µl chromogen substrate was added to each well. After a blue color sufficiently developed, 50 µl stop solution was added to each well and absorbance values at 450 nm were recorded using a microplate reader. A standard curve was generated and used to determine concentrations of kininogen-1 present in the samples analyzed.

To detect serum levels of CEA, a commercially available enzyme immunoassay kit (Whiga, Guangzhou, China) was used according to the manufacturer’s instructions.

### Immunohistochemistry

Kininogen-1 antibody was purchased from Santa Cruz Biotechnology (sc-25799, CA, USA). A previously published protocol was used for the immunohistochemistry assays [20], with kininogen-1 antibody diluted 1∶150. All sections were blindly and independently assessed microscopically by two well-trained pathologists. The intensity of staining was assessed using a semiquantitative scoring system as follows: 0, negative staining; 1, weak staining; 2, moderate staining; and 3, strong staining. The distribution of staining was also graded according to the percentage of stained cells present in the region of interest: 0, positive cells constituted <10% of tumor cells; 1, positive cells constituted 10–50% of tumor cells; 2, positive cells constituted 50–75% of tumor cells; or 3, positive cells constituted >75% of tumor cells. Scores for intensity and distribution were added to provide an overall score for each sample. Briefly, samples receiving zero points were considered negative (0), cases receiving 1–3 points were considered weakly positive (1+), cases receiving 4–7 points were considered moderately positive (2+), and cases receiving a final score >7 were considered strongly positive (3+).

### Statistical Analysis

Statistical analyses were performed using SPSS 13.0. To test the differences between groups, the Chi-square test was applied, with the exception that Student’s t-test was applied to age. Correlations between the immunohistochemical scores determined for kininogen-1 and the clinicopathological parameters of the cohort were evaluated using the Spearman rank-order correlation coefficient. Concentrations of kininogen-1 and CEA were found to have a normal distribution. Therefore, these data were compared using a one-way analysis of variance test. In contrast, multiple comparisons were analyzed using LSD methods. Data were expressed as the mean ± standard error of the mean (SEM). Receiver operating characteristic (ROC) curves were used to determine values of serum kininogen-1 and CEA for the diagnosis of colorectal tumors. The Kaplan-Meier (Log-rank test) was used for survival analysis. For all analyses, a P-value less than 0.05 was considered significant.

## Results

### Proteomic Profiles of ACA and CRC Patient Serum

**Figure 1:**
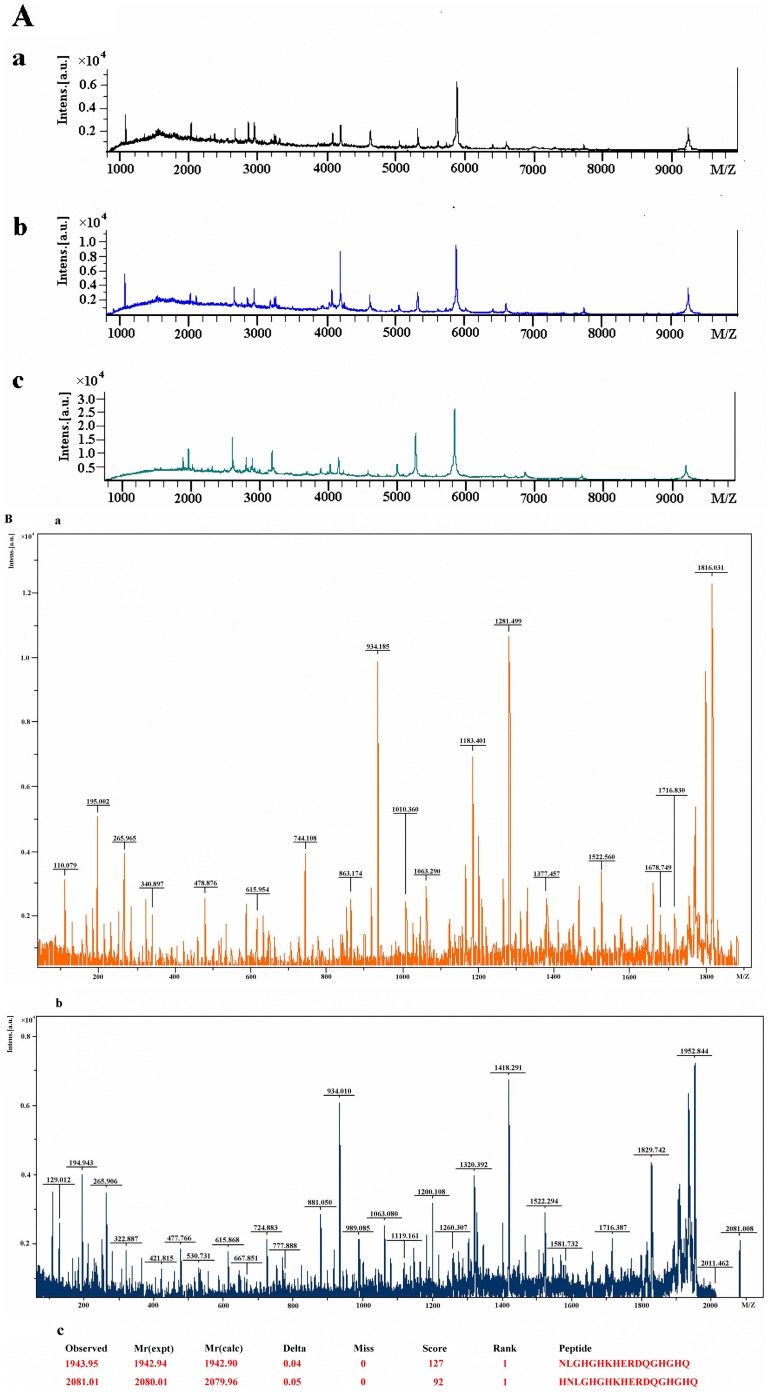
Representative mass spectra of different serum samples and identification of peptides. **A:** Representative mass spectra of control serum (a), serum from a patient with ACA (b), and serum from a patient with CRC (c). **B:** MALDI/MS/MS spectra of the ions at m/z 1943.95 (a) and m/z 2081.01 (b). Panel c provides the results from the Mascot database search, which indicated both peaks correspond to kininogen-1.

A total of 110 sera samples obtained from 35 healthy volunteers (controls), 35 ACA patients, and 40 preoperative CRC patients were subjected to Clinprot analysis. The main clinico-pathological characteristics of patients are showed in **Table S1**. Representative spectra for each of these groups are shown in **Fig. 1A**, and differences in peak position and peak intensity can be observed. Using MALDI-TOF analysis, 70 distinguishable peaks in the 1,000 to 10,000 m/z range were identified between sera samples from controls and ACA patients, and 43 of these peaks were statistically significant (P<0.01,details in Materials S1). In addition, 61 peaks were distinguished between control sera and CRC patient sera, with 54 of these peaks being statistically significant (P<0.01,details in Materials S2).

Using every fifth peak, GA was able to generate cross-validated classification models for the different groups. The best prediction models (details in **Table 1**) achieved a recognition capacity of 98.96%, 100.00%, and 100.00% for comparisons of CRC and control, ACA and control, and ACA and CRC, respectively. Moreover, the cross-validity estimates were 95.49%, 100.00%, and 98.19%, respectively.

**Table 1:** Detected peaks used to generate cross-validated classification models between different groups.

GroupComparisons	Mass-to-charge ratio(m/z)	CrossValidation (%)	RecognitionCapacity (%)
Control versus CRC	6037.33 1347.15 5753.37	95.94	98.96
	1530.03 3192.32		
Control versus ACA	4054.72 2365.93 1529.99	100.00	100.00
	2660.35 2021.19		
ACA versus CRC	4644.29 6088.98 1074.23	98.19	100.00
	4963.96 4248.41		

CRC = colorectal cancer; ACA = advanced colorectal adenoma; Cross Validation and Recognition Capacity were calculated from GA.

### Identification of CRC Markers

A higher concentration of peptides with m/z values of 1943 and 2081 were evident in CRC spectra, yet not in control spectra (*P*<0.01). Using MALDI-TOF/TOF-MS and the Mascot database (**Fig. 1B**), MS/MS fragmentation of these two peptides identified the following sequences, NLGHGHKHERDQGHGHQ and HNLGHGHKHERDQGHGHQ, respectively. Moreover, these peptides were found to represent regions of the same kininogen-1 precursor, a2-thiol proteinase inhibitor.

### Serum Concentrations of Kininogen-1 and CEA for the Different Sera Groups

To validate the expression of kininogen-1 detected in sera from patients with CRC, serum ELISA assays were performed using 366 sera obtained from 85 healthy volunteers, 80 ACA patients, 143 preoperative CRC patients, and 58 postoperative CRC patients. Age, gender, serum kininogen-1 concentrations and CEA concentrations among different groups were shown in **Table 2**. The mean concentrations of kininogen-1 detected in controls and preoperative CRC patients were 153.22±8.43 µg/ml and 215.62±7.63 µg/ml, respectively, with the latter being significantly higher (*P = *0.000). In addition, levels of kininogen-1 were significantly lower in CRC patients following surgery (188.04±11.70 µg/ml; *P = *0.044). For ACA patients, the mean kininogen-1 concentration detected was 194.26±10.14 µg/ml, which was significantly higher than that of the control group (*P = *0.003). In contrast, there was no significant difference between kininogen-1 concentrations detected for preoperative CRC patients and ACA patients (*P = *0.082).

**Table 2:** Serum levels of kininogen-1 and CEA detected for the different groups.

	Controlgroup(n = 85)	ACA patients(n = 80)	CRC patients(preoperative)(n = 143)	CRC patients(postoperative)(n = 58)	*P* value
Gender					
(male/female)	43/42	44/36	83/60	30/28	0.077
Mean age					0.769^a^
(years)	52.51±1.19	51.95±1.23	54.71±1.02	53.97±1.55	0.105^b^
					0.186^c^
					0.695^d^
Kininogen-1					0.003^a^
(µg/ml)	153.22±8.43	194.26±10.14	215.62±7.63	188.04±11.70	0.082^b^
					0.000^c^
					0.044^d^
CEA					0.797^a^
(µg/l)	2.43±0.28	3.10±1.15	14.66±2.25	4.47±0.72	0.000^b^
					0.000^c^
					0.000^d^

CRC = colorectal cancer; ACA = advanced colorectal adenoma. a: N *vs.* ACA; b: ACA *vs.* CRC (preoperative); c: N *vs.* CRC (preoperative); d: CRC (preoperative) *vs.* CRC (postoperative).

For serum CEA concentrations, levels for preoperative CRC patients, postoperative CRC patients, ACA patients, and controls were 14.66±2.25 µg/l, 4.48±0.72 µg/l, 3.10±1.15 µg/l, and 2.43±0.28 µg/l, respectively (ACA *vs.* healthy control, *P = *0.797; CRC *vs.* healthy control, *P = *0.000).

### Diagnostic Value of Serum Kininogen-1 and CEA for Colorectal Tumors

**Figure 2:**
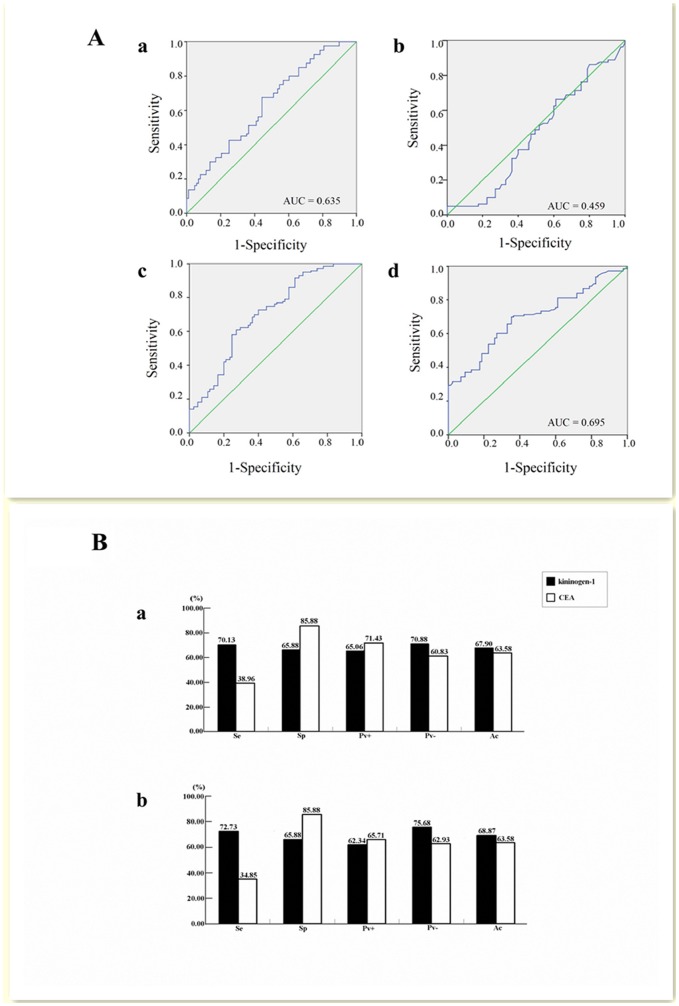
The diagnostic values of serum kininogen-1 and CEA for patients with colorectal tumors. **A:** The ROC curve for serum kininogen-1 (a) and CEA (b) for a diagnosis of ACA, and the ROC curve for serum kininogen-1 (c) and CEA (d) for a diagnosis of CRC. **B:** A comparison of the sensitivity (Se), specificity (Sp), positive predictive value (Pv+), negative predictive value (Pv-), and accuracy (Ac) rates for detection of kininogen-1 and CEA for a diagnosis of Duke’s stage A and B CRC (a) or Duke’s stage C and D CRC (b).

To evaluate the diagnostic value of kininogen-1, a ROC curve analysis was performed. As shown in **Fig. 2** **Aa**, the area under the ROC curve (AUC) for serum kininogen-1 in association with a diagnosis of ACA was 0.635 (95% CI: 0.551–0.719, P = 0.003), while the AUC associated with a diagnosis of CRC was 0.706 (95% CI: 0.635–0.777, P = 0.000; **Fig. 2** **Ac**). Based on these ROC curves, a serum kininogen-1 concentration of 162.99 µg/ml was selected as the optimal cutoff value for differentiating ACA patients and controls, with sensitivity, specificity, positive and negative predictive values, and accuracy rates being 51.25%, 63.53%, 56.94%, 58.06%, and 57.58%, respectively. Similarly, a serum kininogen-1 concentration of 173.96 µg/ml was selected as the optimal cutoff value for differentiating CRC patients and controls, with the associated sensitivity, specificity, positive and negative predictive values, and accuracy rates being 63.64%, 65.88%, 75.83%, 51.85%, and 64.47%, respectively.

The AUC for serum CEA as a diagnosis of ACA was 0.459 (95% CI: 0.370–0.547, P = 0.358; **Fig. 2** **Ab**), and as a diagnosis of CRC was 0.695 (95% CI: 0.627–0.767, P = 0.000; **Fig. 2** **Ad**). The widely accepted cutoff value of 5 µg/l for serum CEA was used in this study since the cutoff value calculated from the ROC curve was 5.095 µg/L. Therefore, using this cutoff value for differentiating CRC patients and controls, the sensitivity, specificity, positive and negative predictive values, and accuracy rates were found to be 38.46%, 85.88%, 82.09%, 45.34%, and 56.14%, respectively. Thus, CEA was associated with lower rates of sensitivity and accuracy compared with kininogen-1.

The aforementioned five parameters were also used to assess kininogen-1 and CEA detection for Duke’s stage A and B CRC patients. For kininogen-1, the rates were 70.13%, 65.88%, 65.06%, 70.88%, and 67.90%, respectively. For CEA, the rates were 38.96%, 85.88%, 71.43%, 60.83%, and 63.58%, respectively. Except for specificity and the positive predictive values, the values of the other parameters associated with kininogen-1 were better than those for CEA (**Fig. 2** **Ba**). When Duke’s stage C and D CRC patients were analyzed, the aforementioned five parameters for kininogen-1 were 72.73%, 65.88%, 62.34%, 75.68%, and 68.87%, respectively, and for CEA, were 34.85%, 85.88%, 65.71%, 62.93%, and 63.58%, respectively. Similar to the Duke’s stage A and B CRC patients, the parameters for kininogen-1, with the exception of specificity and positive predictive values, were better than those for CEA (**Fig. 2** **Bb**). In addition, sensitivity, negative predictive values, and accuracy rates were improved when CRC patients had a positive result for serum kininogen-1 and/or serum CEA. In contrast, specificity rates associated with these positive tests substantially decreased (**Table 3**).

**Table 3:** The diagnosis value of serum kininogen-1 and CEA for CRC.

	Kininogen-1(cutoff 173.96 µg/ml)	CEA(cutoff 5 µg/l)	Kininogen-1or CEA#	Kininogen-1and CEA&
Sensitivity (%)	63.64	38.46	79.72	21.68
Specificity (%)	65.88	85.88	58.82	92.94
Positive predictive values (%)	75.83	82.09	76.51	83.78
Negative predictive values (%)	51.85	45.34	63.29	41.36
Accuracy (%)	64.47	56.14	71.93	48.25

# Positive by either kininogen-1 or CEA serology.

& Positive by both kininogen-1 and CEA serology.

### Expression Levels of Kininogen-1 in Normal Colorectal Mucosa, ACA Tissues, and CRC Tissues

**Figure 3:**
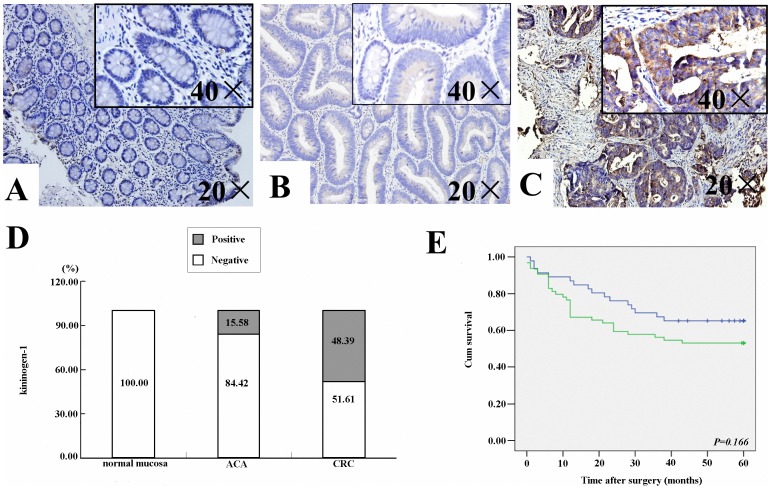
Expression of kininogen-1 in different colorectal tumor tissues and its relationship with survival. Representative immunostainings of kininogen-1 in normal colorectal mucosa (**A**), ACA tissue (**B**), and CRC tissue (**C**). Both overview (20×) and magnification (40×) images are provided, with the latter provided as inset boxes in the top left corner of each panel. **D:** Quantification of kininogen-1 levels in the control, ACA, and CRC groups. **E:** Survival curves for control, ACA, and CRC groups. The blue line represents CRC patients negative for kininogen-1 expression and the green line represents CRC patients positive for kininogen-1 expression.

Kininogen-1 can be detected in the blood under physiological conditions. However, it remains unclear whether higher levels of kininogen-1 detected in the serum of patients with colorectal tumors derives from the colorectal tumor itself. Therefore, immunohistochemistry assays were performed to evaluate expression of kininogen-1 in colorectal tissues. A total of 75 normal colorectal mucosa tissues, 77 ACA tissues, and 248 CRC tissues were examined. No significant difference in the expression of kininogen-1 was found between males versus females among the three groups (*P*>0.05). Furthermore, immunoreactivity for kininogen-1 was found in the cytoplasm of ACA and CRC cells (**Fig. 3** **A–C**), and the expression level of kininogen-1 was significantly higher in CRC tissues than that in ACA tissues or normal mucosa (48.39% *vs.* 15.58% *vs.* 0%, *P*<0.05; **Fig. 3D**).

### Correlation of Kininogen-1 Expression with Clinicopathological Features of ACA and CRC Patients

Correlations between cytoplasmic kininogen-1 levels and clinicopathological features of ACA and CRC patients were analyzed separately. While cytoplasmic accumulation of kininogen-1 was found to negatively correlate with tissue histology for ACA patients (*r_s_* = -0.250, P = 0.029), it did not correlate with tumor location, tumor size, or grade of intraepithelial neoplasia (all *P*>0.05, details in **Table S2**). In contrast, cytoplasmic accumulation of kininogen-1 significantly correlated with Duke’s stage and lymph node metastasis status for CRCs (*r_s_* = 0.151, P = 0.018 and *r_s_* = 0.128, P = 0.045, respectively). However, it did not correlate with tumor location, tumor size, tumor cell differentiation, or distant metastasis (all *P*>0.05, details in **Table S3**).

### Survival Analysis

CRC patients (n = 110) were further analyzed to evaluate a possible association between kininogen-1 immunoreactivity and patient survival. In **Figure 3E**, the survival curve according to cytoplasmic kininogen-1 levels is shown. The mean survival time for CRC patients with negative versus positive kininogen-1 expression was 45.21±3.17 months and 38.15±3.07 months, respectively. Thus, patients with negative kininogen-1 expression had a longer survival period than those with positive kininogen-1 expression, although the difference was not significant (*P = *0.166).

## Discussion

Screening for adenomas and early-stage CRC has decreased the incidence and mortality for CRC in the U.S. over the past few decades [3]. However, current screening methods do not provide good sensitivity. Therefore, efforts are continuing to be directed towards developing novel diagnostic or screening serum markers for CRC. In addition, advances in proteomics technology have facilitated the identification of novel biomarkers. In particular, Clinprot technology has been found to provide highly accurate and reproducible results, a good level of sensitivity, and is compatible with a high throughput format for the rapid identification of proteins [21]. Consequently, proteomic profiles for various human diseases have been obtained using Clinprot methodology [22]–[24]. In the present study, the Clinprot protocol provided predicting models for CRC and ACA versus healthy controls, and also between ACA and CRC. Moreover, the recognition capacities of these models were 98.96%, 100.00%, and 100.00%, respectively. Thus, kininogen-1 was identified as a potential marker of CRC and ACA, and these results were validated using ELISA. Taken together, these results confirm the accuracy of Clinprot technology.

Accumulating evidence continues to demonstrate a role for kininogen-1 in carcinogenesis [25]. For example, significantly reduced levels of kininogen-1 have been detected in the urine of patients with ovarian carcinoma compared with control subjects [26]. Previous studies have also demonstrated that kininogen-1 exhibits antiangiogenic properties and mediates inhibitory actions on the proliferation of endothelial cells [27]. Furthermore, in cancer patients, lower levels of kininogen-1 expression have been detected in blood samples, and these levels may contribute to the survival of the cancer cells present [28]. However, the role of kininogen-1 in carcinogenesis, especially in CRC, has remained unclear. In the present study, serum kininogen-1 levels in patients with ACA or CRC were found to be significantly higher compared with the healthy controls. It is widely accepted that ACA is a precancerous lesion of CRC [29]. Thus, a significant increase in serum kininogen-1 levels in these patients may represent a marker for the early detection of CRC, and this is consistent with the results of Qiu *et al.* [30]. Although, in a study of kininogen-1 expression detected in 118 plasma samples obtained from patients with gastrointestinal cancer, significantly lower levels of kininogen-1 were detected [31]. While this is in contrast with the results of the present study, this difference may be due to differences in sample size, sample resources, and the detection method used.

Measurements of CEA levels have been found to be unsuitable for population screenings due to the lack of sensitivity of this assay in the early stages of CRC [32], [33]. A panel of the American Society of Clinical Oncology has also recommended against CEA testing for CRC screening [34]. Correspondingly, the CEA assays performed in the present study showed no diagnostic value for ACA (AUC = 0.453), and also exhibited a lower sensitivity (38.96% *vs.* 70.13%) and accuracy (63.58% *vs.* 67.90%) for Duke’s stage A and B CRC, compared with kininogen-1. These results indicate that monitoring serum levels of kininogen-1 is more valuable than detecting CEA in the early stages of CRC. Moreover, when both serum levels of kininogen-1 and levels of CEA were monitored in CRC patients, the specificity and positive predictive value of these results improved. Therefore, detection of kininogen-1 in combination with other tumor markers is recommended. In addition, if a patient is found to be positive for kininogen-1 and CEA, yet negative for alpha fetal protein, then it is possible he/she suffers from CRC rather than liver cancer. Further studies will be needed to confirm this hypothesis.

In postoperative CRC patients, serum levels of kininogen-1 were lower than those of preoperative CRC patients. Although it remains unclear why this was observed, it is hypothesized that increased production of kininogen-1 derives from tumor tissues. This is supported by the immunohistochemistry results obtained that showed kininogen-1 accumulated in the cytoplasm of colorectal tumor cells. However, the mechanistic details of this process remain unclear. In addition, cytoplasmic accumulation of kininogen-1 significantly correlated with lymph node metastasis status in patients with CRC. However, a survival analysis of CRC patients according to kininogen-1 expression found no significant difference between negative and positive kininogen-1 expression group, indicating the prognosis value of kininogen-1 is limited.

To our knowledge, this is the first study to report the detection of kininogen-1 in ACA and CRC patients, with validation by ELISA and immunohistochemistry. Based on these results, kininogen-1 appears to be a potential CRC marker, which may be valuable for the early detection of CRC, particularly in combination with other biomarkers in population screenings for CRC. An analysis of additional patients, including standardized processing of the samples obtained, is needed to verify and expand the present results. Moreover, mechanistic studies of kininogen-1 in colorectal tumors are warranted.

## Supporting Information

Table S1 The main clinicopathological characteristics of patients included in the discovery and validation cohorts.
(DOC)

Table S2 Correlation between kininogen-1 expression and clinicopathologic features of ACA patients.
(DOC)

Table S3 Correlation between kininogen-1 expression and the clinicopathologic features of CRC patients.
(DOC)

Materials S1 ClinProt Peak Statistic between healthy control and ACA patients.
(XLS)

Materials S2 ClinProt Peak Statistic between healthy control and CRC patients.
(XLS)
