# Supplementary material for: Identification of Kininogen-1 as a Serum Biomarker for the Early Detection of Advanced Colorectal Adenoma and Colorectal Cancer
Source: PLoS One. 2013 Jul 23;8(7):e70519. doi: 10.1371/journal.pone.0070519 (PMC3720899; doi:10.1371/journal.pone.0070519)
Supplement: Table S1 — The main clinicopathological characteristics of patients included in the discovery and validation cohorts. (DOC) [file pone.0070519.s001.doc]

**Table S1**. The main clinicopathological characteristics of patients included in the discovery and validation cohorts.

|  | Discovery Cohort | | | | Validation Cohort | | | |
| --- | --- | --- | --- | --- | --- | --- | --- | --- |
|  | Control | ACA | CRC | P value | Control | ACA | CRC | P value |
|  | group | patients | patients |  | group | patients | patients |  |
|  | (n = 35) | (n = 35) | (n = 40) |  | (n = 85) | (n = 80) | (n = 143) |  |
| Mean age | 51.40 ± | 52.86 ± | 56.78 ± | 0.583^A^ | 52.51 ± | 51.95 ± | 54.71 ± | 0.769^a^ |
| (years) | 2.02 | 1.70 | 1.96 | 0.140^B^ | 1.19 | 1.23 | 1.02 | 0.105^b^ |
|  |  |  |  | 0.061^C^ |  |  |  | 0.186^c^ |
| Gender | 21/14 | 19/16 | 25/15 | 0.057 | 43/42 | 44/36 | 83/60 | 0.068 ^b^ |
| (male/female) |  |  |  |  |  |  |  |  |
| Tumor location |  |  |  | 0.077 ^B^ |  |  |  | 0.059 |
| Proximal colon | --- | 9 | 8 |  | --- | 21 | 39 |  |
| Distal colon | --- | 12 | 13 |  | --- | 26 | 48 |  |
| Rectum | --- | 14 | 19 |  | --- | 33 | 56 |  |
| TNM stage |  |  |  |  |  |  |  |  |
| I | --- | --- | 8 | --- | --- | --- | 14 | --- |
| II | --- | --- | 12 |  | --- | --- | 63 |  |
| III | --- | --- | 11 |  | --- | --- | 37 |  |
| IV | --- | --- | 9 |  | --- | --- | 29 |  |

CRC = colorectal cancer; ACA= advanced colorectal adenoma.

A: Controls vs. ACA in the discovery cohort; B: ACA *vs.* CRC in the discovery cohort; C: Controls *vs.* CRC in the discovery cohort.

a: Controls *vs.* ACA in the validation cohort; b: ACA *vs.* CRC in the validation cohort; c: Controls *vs.* CRC in the validation cohort.

Student’s t-test was used for age, and the Chi-square test was used for gender and location.
