# Supplementary material for: Identification of Kininogen-1 as a Serum Biomarker for the Early Detection of Advanced Colorectal Adenoma and Colorectal Cancer
Source: PLoS One. 2013 Jul 23;8(7):e70519. doi: 10.1371/journal.pone.0070519 (PMC3720899; doi:10.1371/journal.pone.0070519)
Supplement: Table S2 — Correlation between kininogen-1 expression and clinicopathologic features of ACA patients. (DOC) [file pone.0070519.s002.doc]

**Table S2.**  Correlation between kininogen-1 expression and clinicopathologic features of ACA patients.

|  | Kininogen-1 expression | | | | |  | |
| --- | --- | --- | --- | --- | --- | --- | --- |
|  | 0 | 1+ | 2+ | 3+ | ***rs*** | | *P* value |
| Tumor location |  |  |  |  | -0.155 | | 0.180 |
| Proximal colon | 17 | 3 | 0 | 1 |  | |  |
| Distal colon | 20 | 3 | 3 | 0 |  | |  |
| Rectum | 28 | 1 | 1 | 0 |  | |  |
| Tumor size |  |  |  |  | -0.158 | | 0.171 |
| < 30 mm | 48 | 6 | 4 | 1 |  | |  |
| ≥ 30 mm | 17 | 1 | 0 | 0 |  | |  |
| Tissue histology |  |  |  |  | -0.250 | | 0.029 |
| Tubular | 19 | 4 | 2 | 1 |  | |  |
| Tubulovillous | 36 | 3 | 2 | 0 |  | |  |
| Villotubular | 10 | 0 | 0 | 0 |  | |  |
| Grade of intraepithelial neoplasia |  |  |  |  | 0.008 | | 0.943 |
| Low-grade | 32 | 4 | 1 | 1 |  | |  |
| High-grade | 33 | 3 | 3 | 0 |  | |  |
