# Supplementary material for: Identification of Kininogen-1 as a Serum Biomarker for the Early Detection of Advanced Colorectal Adenoma and Colorectal Cancer
Source: PLoS One. 2013 Jul 23;8(7):e70519. doi: 10.1371/journal.pone.0070519 (PMC3720899; doi:10.1371/journal.pone.0070519)
Supplement: Table S3 — Correlation between kininogen-1 expression and the clinicopathologic features of CRC patients. (DOC) [file pone.0070519.s003.doc]

**Table S3.**  Correlation between kininogen-1 expression and the clinicopathologic features of CRC patients.

|  | Kininogen-1 expression | | | |  | | |
| --- | --- | --- | --- | --- | --- | --- | --- |
|  | 0 | 1+ | 2+ | 3+ | ***rs*** | *P* value |  |
| Dukes’ stage |  |  |  |  | 0.151 | 0.018 |  |
| A | 36 | 13 | 3 | 1 |  |  |  |
| B | 50 | 38 | 10 | 3 |  |  |  |
| C | 27 | 25 | 7 | 4 |  |  |  |
| D | 15 | 12 | 2 | 2 |  |  |  |
| TNM-T |  |  |  |  | 0.091 | 0.154 |  |
| 1 | 9 | 6 | 0 | 0 |  |  |  |
| 2 | 23 | 10 | 5 | 1 |  |  |  |
| 3 | 74 | 52 | 12 | 7 |  |  |  |
| 4 | 22 | 20 | 5 | 2 |  |  |  |
| TNM-N |  |  |  |  | 0.128 | 0.045 |  |
| 0 | 81 | 48 | 13 | 4 |  |  |  |
| 1 | 34 | 21 | 5 | 2 |  |  |  |
| 2 | 13 | 19 | 4 | 4 |  |  |  |
| TNM-M |  |  |  |  | -0.006 | 0.925 |  |
| 0 | 113 | 77 | 21 | 8 |  |  |  |
| 1 | 15 | 11 | 1 | 2 |  |  |  |
| Tumor location |  |  |  |  | -0.041 | 0.522 |  |
| Proximal colon | 16 | 12 | 3 | 2 |  |  |  |
| Distal colon | 32 | 22 | 4 | 5 |  |  |  |
| Rectum | 80 | 54 | 15 | 3 |  |  |  |
| Differentiation |  |  |  |  | -0.008 | 0.899 |  |
| Well | 50 | 37 | 11 | 2 |  |  |  |
| Moderate | 61 | 37 | 9 | 6 |  |  |  |
| Poor | 17 | 14 | 2 | 2 |  |  |  |
| Tumor size |  |  |  |  | 0.066 | 0.299 |  |
| < 30 mm | 9 | 4 | 1 | 0 |  |  |  |
| ≥ 30 mm | 119 | 84 | 21 | 10 |  |  |  |
